# Supplementary material for: Expression of pH-Sensitive GPCRs in Peritoneal Carcinomatosis of Colorectal Cancer—First Results
Source: J Clin Med. 2023 Feb 23;12(5):1803. doi: 10.3390/jcm12051803 (PMC10003041; doi:10.3390/jcm12051803)
Supplement: Supplementary file 1 [file jcm-12-01803-s001.zip › jcm-2155330-supplementary.pdf]

SUPPLEMENTARY INFORMATION

Supplementary Figure S1

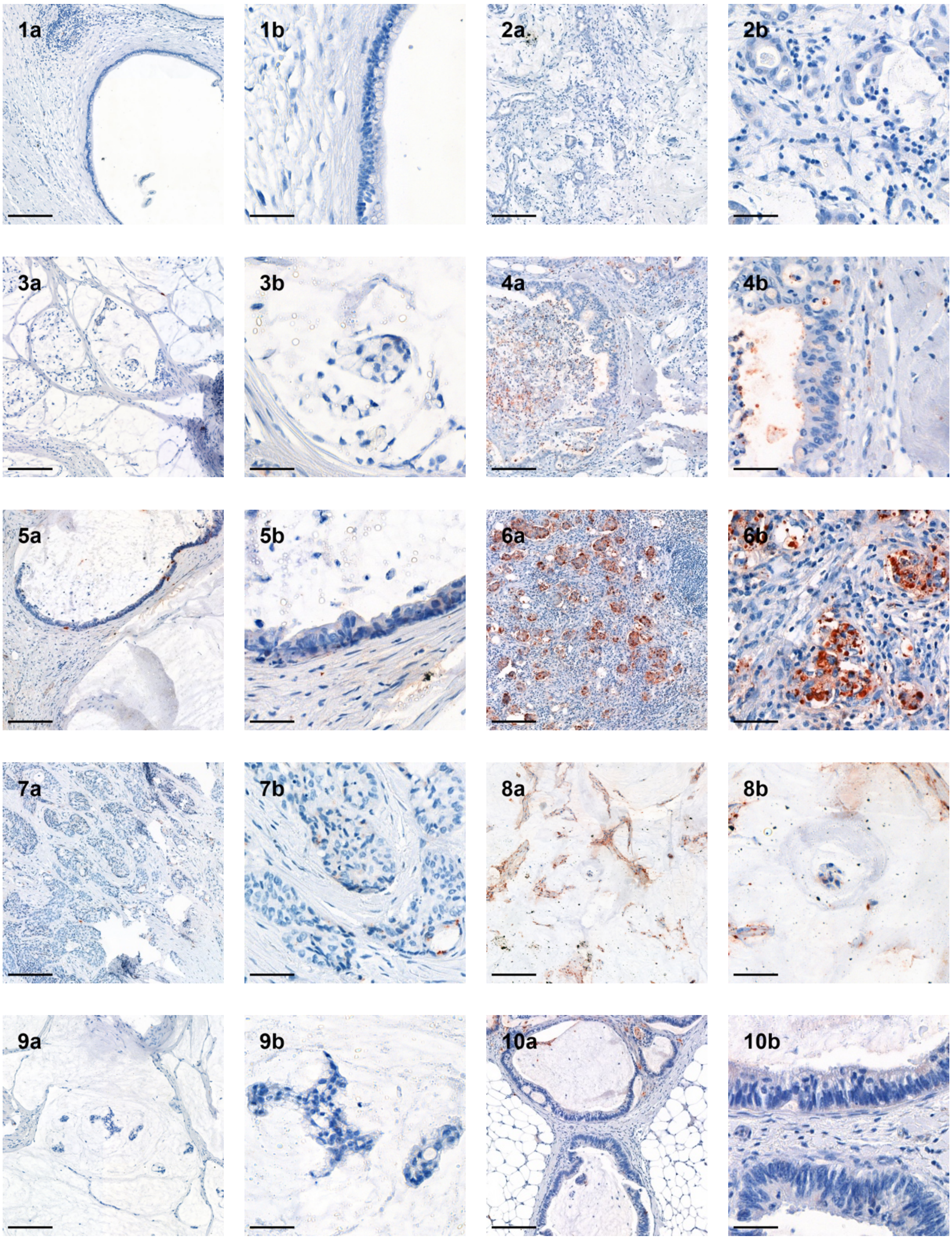

Supplementary Figure S1: Immunohistochemistry for *GPR 4.* The number in each slide corresponds to the patient number (compare also Table 1). “a” represents a 10-fold magnification (scale bar 200 µm) and “b” a 40-fold magnification (scale bar 50 µm).

Supplementary Figure 2

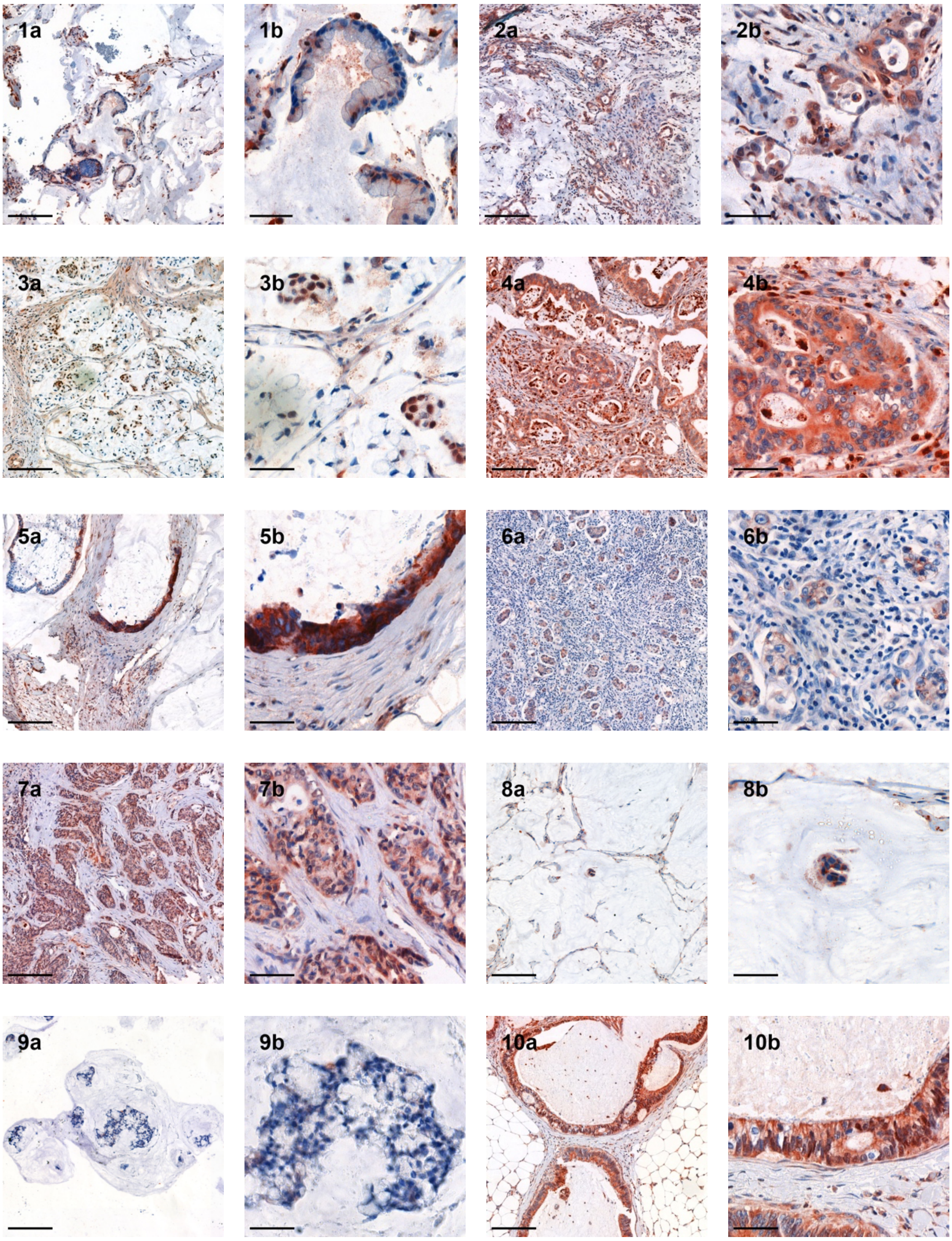

Supplementary Figure S2: Immunohistochemistry for *GPR 65*. The number in each slide corresponds to the patient number (compare also Table 1). “a” represents a 10-fold magnification (scale bar 200 µm) and “b” a 40-fold magnification (scale bar 50 µm).

Supplementary Figure S3

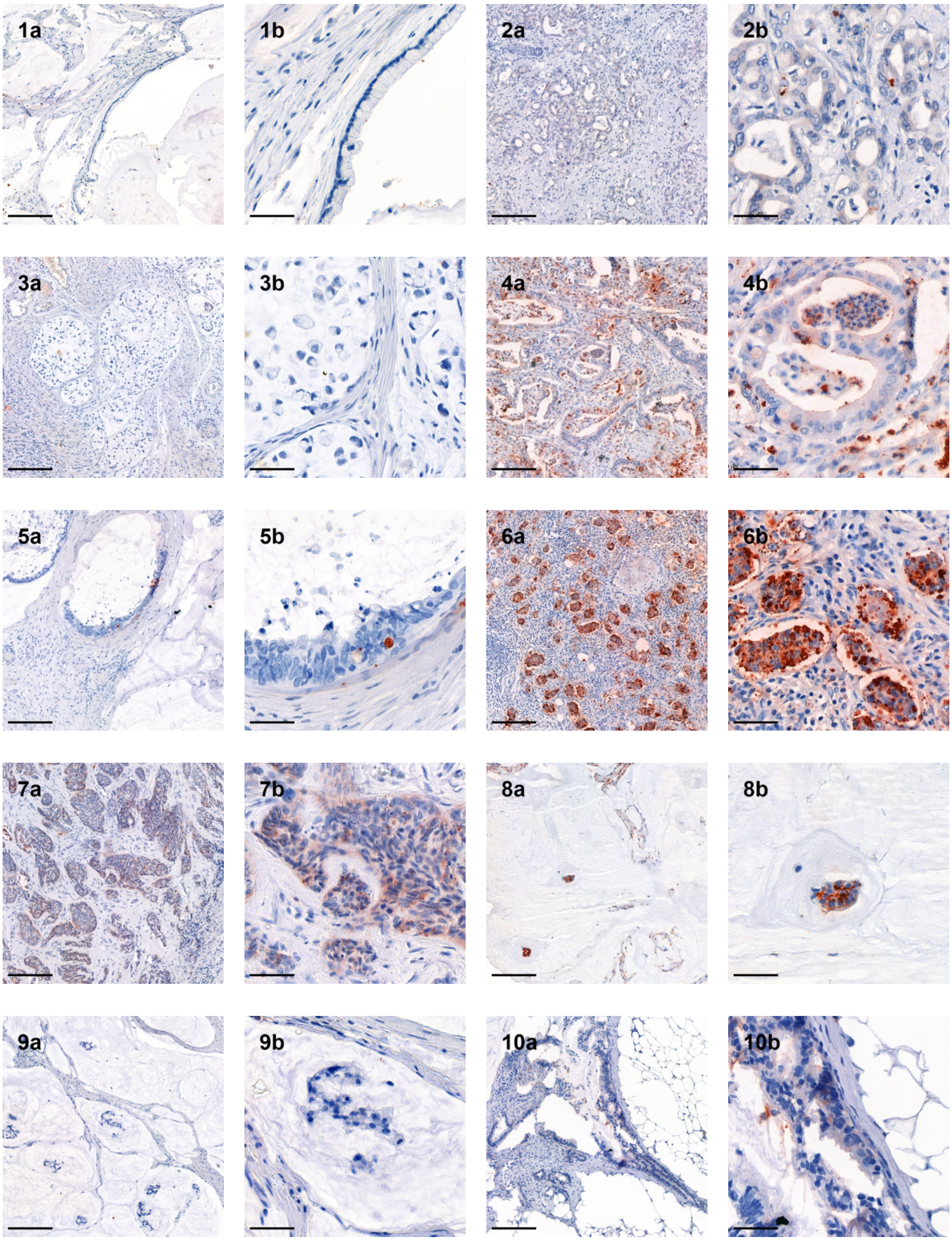

Supplementary Figure S3: Immunohistochemistry for *GPR 68*. The number in each slide corresponds to the patient number (compare also Table 1). “a” represents a 10-fold magnification (scale bar 200 µm) and “b” a 40-fold magnification (scale bar 50 µm).

Supplementary Figure S4

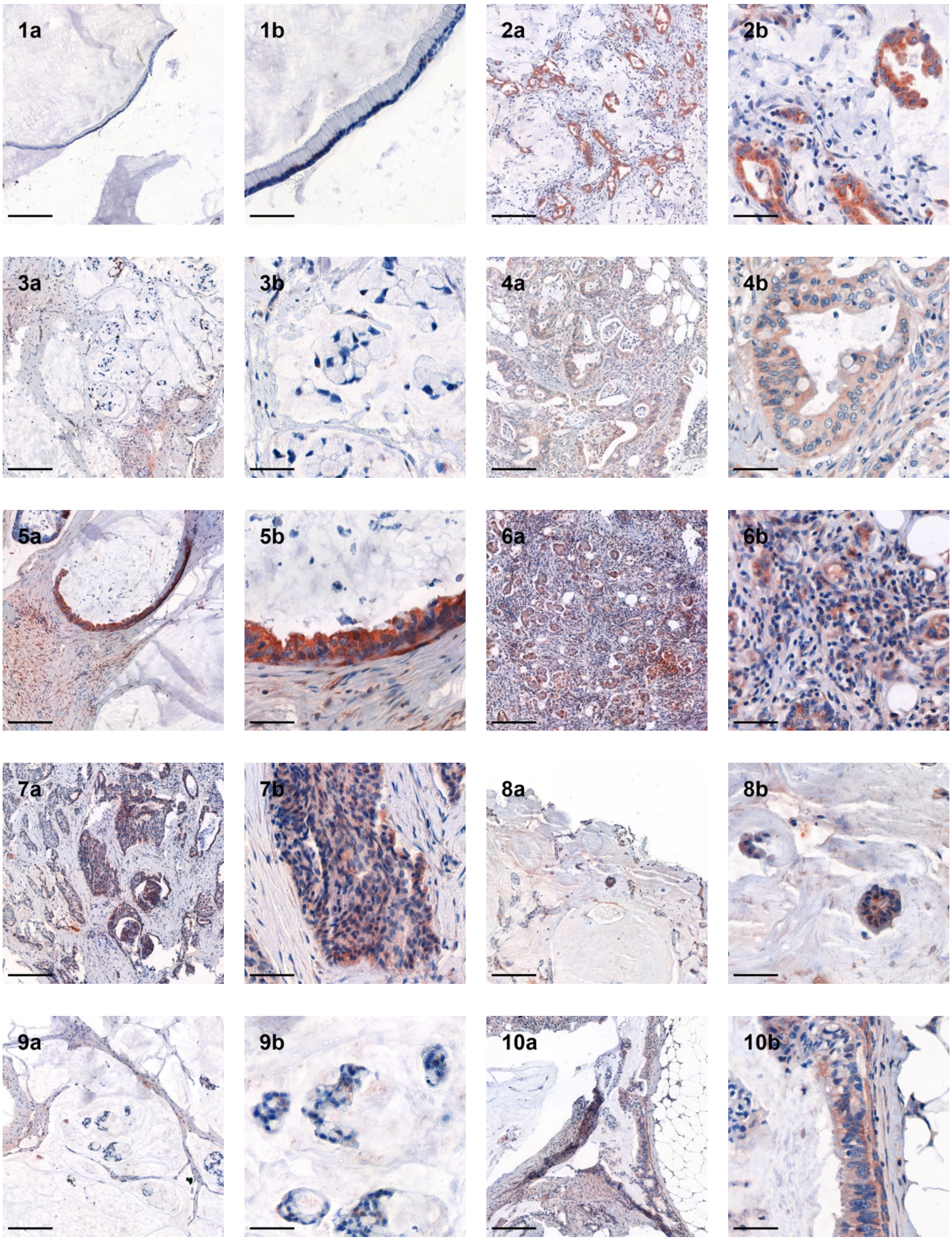

Supplementary Figure S4: Immunohistochemistry for *GPR 132*. The number in each slide corresponds to the patient number (compare also Table 1). “a” represents a 10-fold magnification (scale bar 200 µm) and “b” a 40-fold magnification (scale bar 50 µm).

Supplementary Figure S5

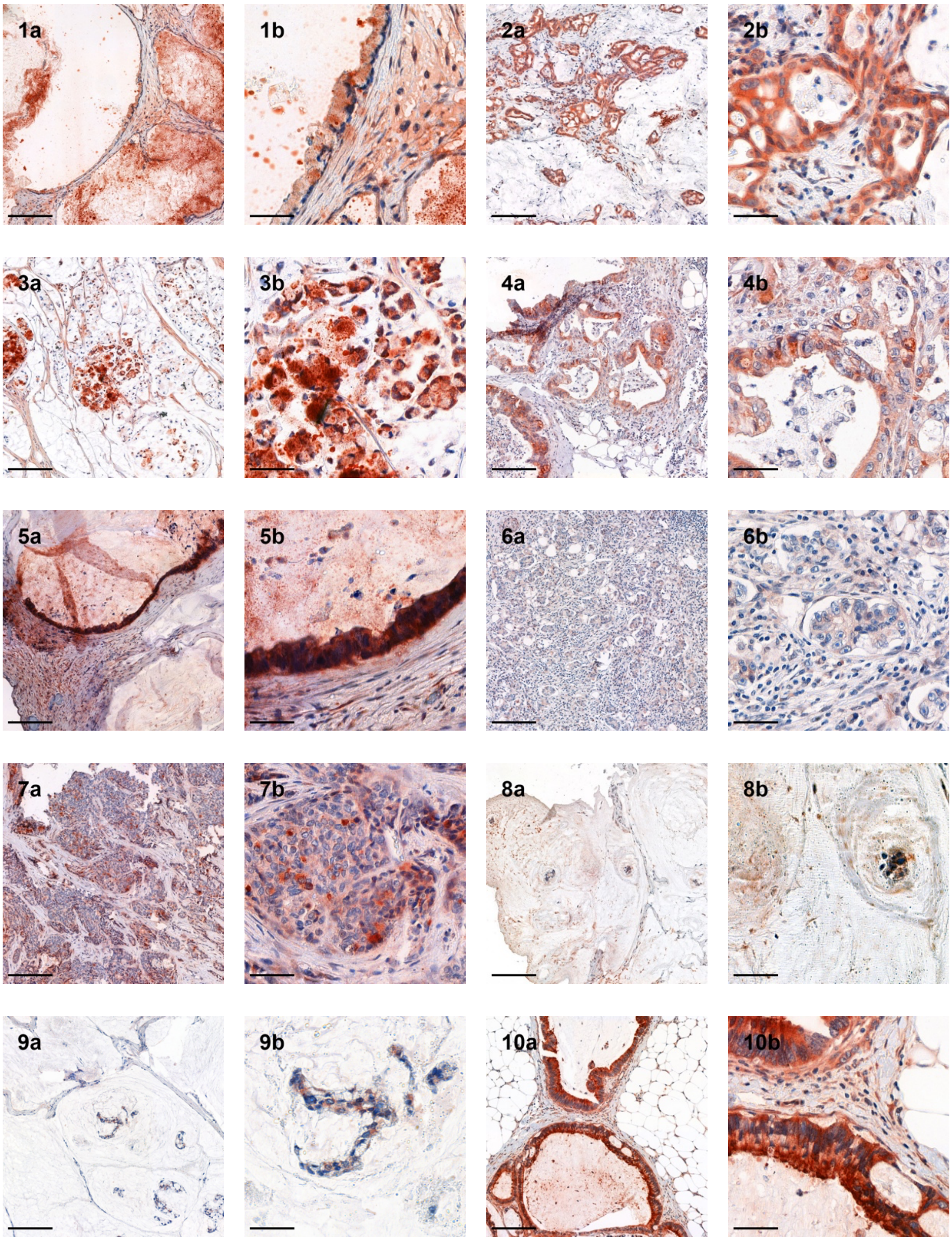

Supplementary Figure S5: Immunohistochemistry for *GPR 151*. The number in each slide corresponds to the patient number (compare also Table 1). “a” represents a 10-fold magnification (scale bar 200 µm) and “b” a 40-fold magnification (scale bar 50 µm).

Supplementary Figure S6

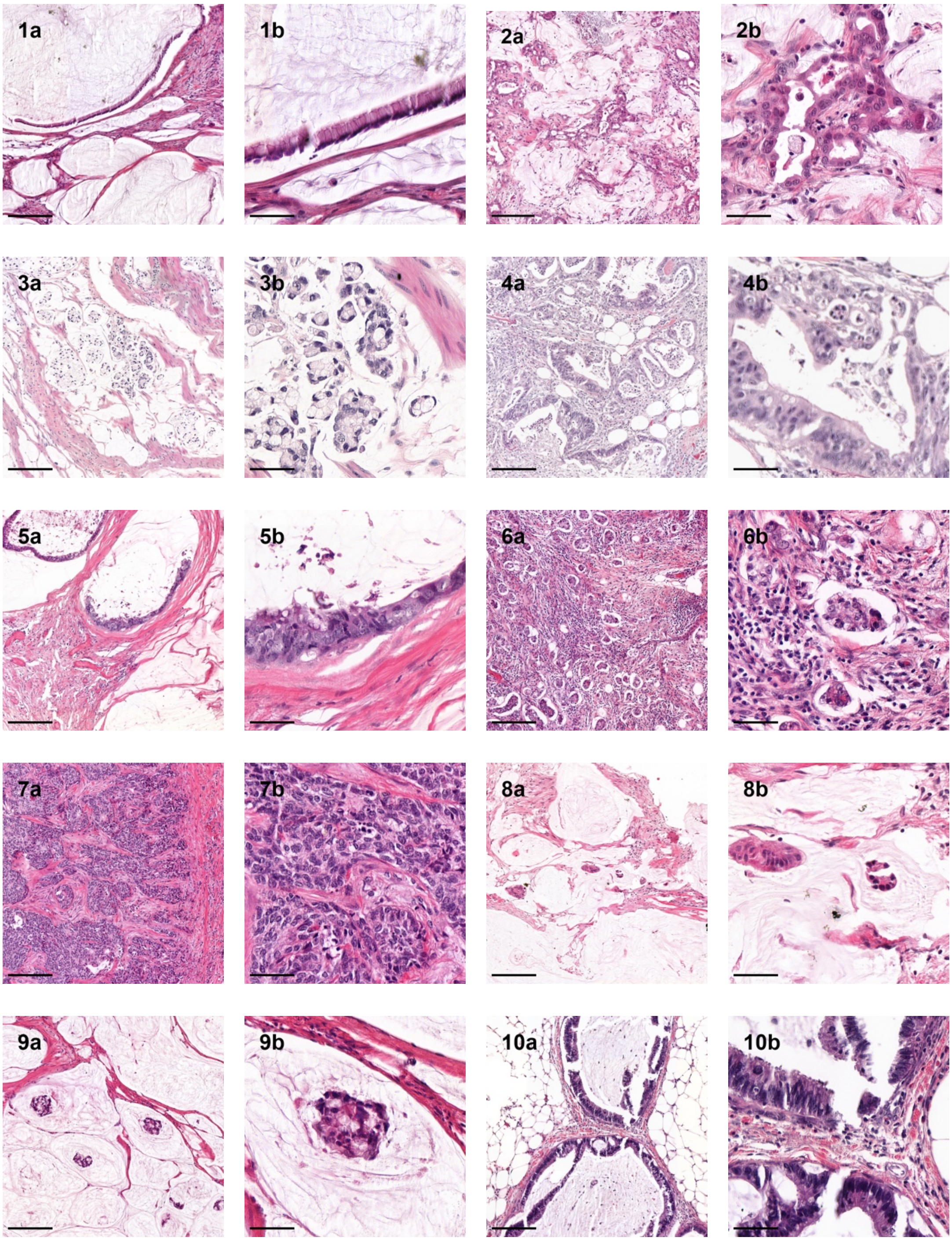

Supplementary Figure S6: ***HE*** **staining.** The number in each slide corresponds to the patient number (compare also Table 1). "a" represents a 10-fold magnification (scale bar 200 µm) and "b" a 40-fold magnification (scale bar 50 µm).
